# Pretreatment DCE-MRI-Based Deep Learning Outperforms Radiomics Analysis in Predicting Pathologic Complete Response to Neoadjuvant Chemotherapy in Breast Cancer

**DOI:** 10.3389/fonc.2022.846775

**Published:** 2022-03-10

**Authors:** Yunsong Peng, Ziliang Cheng, Chang Gong, Chushan Zheng, Xiang Zhang, Zhuo Wu, Yaping Yang, Xiaodong Yang, Jian Zheng, Jun Shen

**Affiliations:** ^1^Division of Life Sciences and Medicine, School of Biomedical Engineering (Suzhou), University of Science and Technology of China, Hefei, China; ^2^Medical Imaging Department, Suzhou Institute of Biomedical Engineering and Technology, Chinese Academy of Sciences, Suzhou, China; ^3^Department of Radiology, Sun Yat-sen Memorial Hospital, Sun Yat-sen University, Guangzhou, China; ^4^Guangdong Provincial Key Laboratory of Malignant Tumor Epigenetics and Gene Regulation, Breast Tumor Center, Sun Yat-sen Memorial Hospital, Sun Yat-sen University, Guangzhou, China

**Keywords:** breast cancer, neoadjuvant chemotherapy, dynamic contrast-enhanced magnetic resonance imaging, radiomics, deep learning

## Abstract

**Purpose:**

To compare the performances of deep learning (DL) to radiomics analysis (RA) in predicting pathological complete response (pCR) to neoadjuvant chemotherapy (NAC) based on pretreatment dynamic contrast-enhanced MRI (DCE-MRI) in breast cancer.

**Materials and Methods:**

This retrospective study included 356 breast cancer patients who underwent DCE-MRI before NAC and underwent surgery after NAC. Image features and kinetic parameters of tumors were derived from DCE-MRI. Molecular information was assessed based on immunohistochemistry results. The image-based RA and DL models were constructed by adding kinetic parameters or molecular information to image-only linear discriminant analysis (LDA) and convolutional neural network (CNN) models. The predictive performances of developed models were assessed by receiver operating characteristic (ROC) curve analysis and compared with the DeLong method.

**Results:**

The overall pCR rate was 23.3% (83/356). The area under the ROC (AUROC) of the image-kinetic-molecular RA model was 0.781 [95% confidence interval (CI): 0.735, 0.828], which was higher than that of the image-kinetic RA model (0.629, 95% CI: 0.595, 0.663; *P* < 0.001) and comparable to that of the image-molecular RA model (0.755, 95% CI: 0.708, 0.802; *P* = 0.133). The AUROC of the image-kinetic-molecular DL model was 0.83 (95% CI: 0.816, 0.847), which was higher than that of the image-kinetic and image-molecular DL models (0.707, 95% CI: 0.654, 0.761; 0.79, 95% CI: 0.768, 0.812; *P* < 0.001) and higher than that of the image-kinetic-molecular RA model (0.778, 95% CI: 0.735, 0.828; *P* < 0.001).

**Conclusions:**

The pretreatment DCE-MRI-based DL model is superior to the RA model in predicting pCR to NAC in breast cancer patients. The image-kinetic-molecular DL model has the best prediction performance.

## Introduction

Breast cancer is the most common diagnosed cancer and the most common cause of cancer death worldwide (1). Neoadjuvant chemotherapy (NAC) has been well established in managing breast cancer for patients with locally advanced cancer and early-stage operable breast cancers of specific molecular subtypes (2). Pathologic complete response (pCR) is mainly used to evaluate the degree of regression after NAC, as pCR has been demonstrated to be associated with better survival (3). However, only 7%–38% of breast cancers can achieve pCR (4). Thus, predicting pCR early before NAC is imperative and can timely switch to a new personalized treatment strategy and exempt from unnecessary chemotherapy toxicity patients with a low possibility of pCR.

MRI has been proven to be most accurate for measuring treatment response based on the change of tumor size or volume (5). Other than morphologic criteria, kinetic parameters including quantitative parameters, e.g., K^trans^ (volume transfer constant), K_ep_ (reverse reflux rate constant), V_e_ (volume fraction of extravascular extracellular space), and V_p_ (volume fraction of plasma), and semiquantitative parameters, e.g., TTP (time to peak), MaxConc (maximum concentration), MaxSlope (maximal slope), and AUC (area under the curve), can be derived from dynamic contrast-enhanced MRI (DCE-MRI), which can reflect tumor microvascular function such as vascular density and permeability (6). It has been reported that reduction in the K^trans^ or K_ep_ after two cycles of NAC is associated with the response to NAC (7, 8). However, only a few studies with small sample sizes have evaluated the power of pretreatment kinetic parameters in predicting pCR, with a reported moderate predictive performance [area under the receiver operating characteristic (AUROC) = 0.56–0.66] (9, 10).

Recently, imaging-based machine learning approaches have been used to predict therapeutic response by quantifying the tumor heterogeneity and irregularity of tissue components (11). Radiomics analysis (RA) and deep learning (DL) are the two most popular machine learning approaches, which have immense capability to obtain minable data by evaluating tumor features of images (11–14). RA relies on a pipeline including extraction of numerous handcrafted imaging features, followed by feature selection and then machine learning-based classification (11). However, the performance of radiomics models derived from pretreatment DCE-MRI is limited in predicting pCR with an AUROC ranging from 0.568 to 0.79 (12, 15, 16). DL can automatically learn discriminative features directly from images without the necessity of feature predefinition (17). The AUROC of DL models developed from pretreatment DCE-MRI alone ranged from 0.553 to 0.7969 (13, 14). In addition, a recent study has shown that the convolutional neural network (CNN) model based on pretreatment DCE-MRI (AUROC = 0.7969) had better prediction performance than the CNN model based on posttreatment DCE-MRI (AUROC = 0.7737) (13). So far, there is a lack of head-to-head comparison of predictive performance between RA and DL models based on pretreatment DCE-MRI in predicting pCR to NAC. Furthermore, whether an integrative model, which incorporates tumor image features, kinetic parameters, and molecular biomarkers, could improve predictive performance remains to be determined.

In this study, women with breast cancer who received NAC were retrospectively included. The image features and kinetic parameters of tumors derived from pretreatment DCE-MRI and molecular information determined by immunohistochemistry (IHC) were used to develop prediction models. The purpose of our study was to determine whether the DL model is better than the RA model in predicting pCR to NAC in breast cancer patients based on pretreatment DCE-MRI and whether incorporating molecular biomarkers and kinetic parameters into image features can improve the predictive performance.

## Materials and Methods

### Study Population

**Figure 1:**
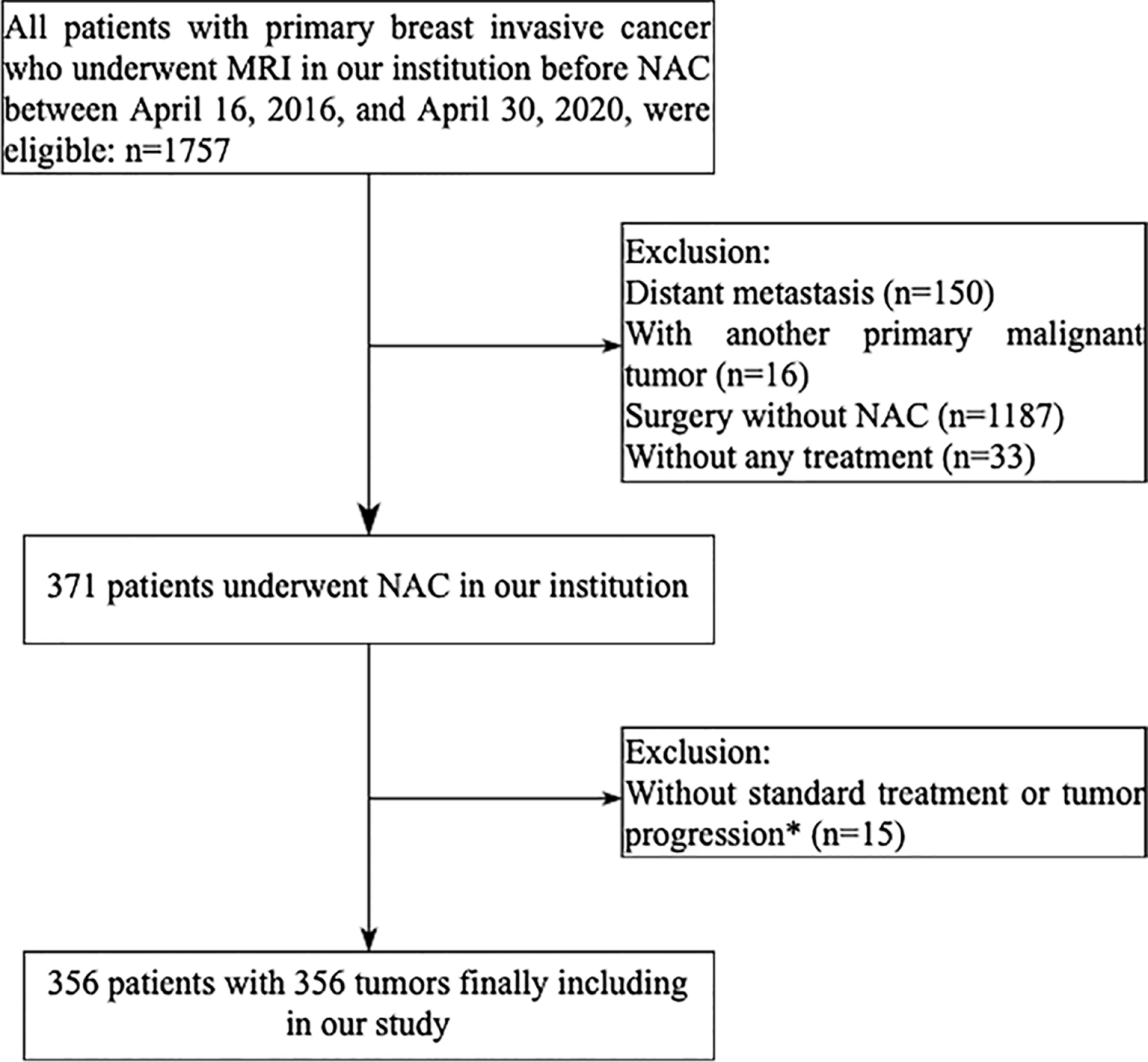
Flowchart of patient enrollment in the study. *Seven patients did not complete the established neoadjuvant chemotherapy program because of tumor progression, three patients did not have an operation, five HER2-positive patients did not receive trastuzumab plus pertuzumab treatment.

This retrospective study was approved by the Ethics Committee of Sun Yat-sen Memorial Hospital, with a waiver for informed consent from all participants. In our institution, a total of 1,757 patients with primary breast invasive cancer were diagnosed between April 16, 2016, and April 30, 2020. The inclusion criteria were as follows: 1) an initial diagnosis of primary invasive breast cancer; 2) DCE-MRI performed before biopsy and within 1 week before NAC; 3) surgical excision of the tumor whether achieving pCR or non-pCR after NAC treatment. The exclusion criteria were distant metastasis (n = 150), another malignant tumor (n = 16), surgery but without NAC (n = 1,187), without any treatment (n = 33), non-standard NAC treatment (n = 8), or tumor progression during NAC (n = 7). The patient enrollment pathway is shown in the consort diagram (**Figure 1**). Finally, 356 patients were included for analysis. The entire cohorts were split into independent training and validation dataset by 5-fold cross-validation (18). Four-fold data (80% of the tumors) were used as training dataset, and the remaining one-fold data (20% of the tumors) were used as validation dataset. The prediction probabilities of five independent validation sets were collected as a whole set and used to evaluate the model performance. The 5-fold cross-validation procedure is illustrated in **Supplementary E1** and **Supplementary Figure S1**.

### MRI Protocol

Breast MRI was performed on a 1.5T unit (Magnetom Avanto; Siemens Medical Solutions, Erlangen, Germany) with patients in the head-first prone position. The body coil was used as the transmitter, and a dedicated 8-channel phased-array breast coil (Siemens Medical Solutions, Erlangen, Germany) was used as the receiver. MRI sequences consisted of axial T2-weighted turbo spin-echo (TSE) with short tau inversion recovery (STIR) sequence; axial T1-weighted volume interpolated body examination (T1W-VIBE) with Dixon sequence, and axial diffusion-weighted imaging (DWI) with spectral attenuated inversion recovery (SPAIR) fat saturation with 2 b values (b = 0, 800 s/mm^2^) and axial DCE imaging. DCE images were acquired by using a 3D fat-suppressed T1W-VIBE sequence. The DCE acquisition consisted of 40–70 measurements with a temporal resolution of 8 s and a total of 5–7 min of imaging time. After two consecutive measurements, gadodiamide (Gd-DTPA-BMA) (Omniscan; GE Healthcare, Ireland) was administered *via* intravenous bolus injection at a dosage of 0.1 mmol/kg and a flow rate of 3.5 ml/s, followed by a 20-ml saline flush. Before DCE acquisition, multiple flip angle images (2°, 4°, 6°, 8°, 10°, and 12°) were obtained for the calculation of T1 maps using the same sequence and parameters except for the flip angle. The details of acquisition parameters of MRI pulse sequence are provided in **Supplementary Table S1**.

### Neoadjuvant Chemotherapy Programs and Outcome

The diagnosis of all patients was established by a core needle biopsy of the primary tumor before NAC. The regimens of NAC, provided in **Supplementary E2**, were defined according to the National Comprehensive Cancer Network (NCCN) guideline (19). According to the Food and Drug Administration criteria (20), all patients underwent surgical resection of the tumors and sentinel lymph node dissection (SLNB) or axillary lymph node dissection (ALND) after NAC. The resected tumors and lymph nodes were sampled for histologic examination to evaluate the chemotherapeutic response. The pCR (ypT0/Tis-ypN0) was defined as the absence of residual invasive tumor in the breast and axillary lymph nodes on the operative specimen (breast tumor and axillary lymph nodes) following NAC. In contrast, non-pCR was defined as a residual invasive cancer in the breast or axillary nodes.

### Kinetic Parameters and Prediction Model Building

DCE-MRI data were analyzed independently by two radiologists (ZC and CZ with 10 years and 8 years of experience with breast MRI) using specialized quantitative analysis software (Omni Kinetics, GE Healthcare). The kinetic parameters were calculated using the extended Tofts model. During measurement, the regions of interest (ROIs) were carefully drawn to cover the whole tumor. Necrotic or cystic areas of the lesions, if presented, were excluded from the evaluation. The intraclass correlation coefficient (ICC) of kinetic parameters between the two readers was 0.834–0.977. Data from the two readers were averaged for analysis. The least absolute shrinkage and selection operator (LASSO) regression analysis was applied to select independent predictive kinetic parameters. These selected kinetic parameters were used to construct the kinetic-only RA model using a robust supervised classifier, linear discriminant analysis (LDA) (21), which was employed to classify the NAC treatment efficiency by searching for a linear combination of the independent predictive kinetic parameters. A multilayer perceptron (MLP) neural network (22) was employed to construct the kinetic-only DL model. The structure of the MLP neural network is shown in **Supplementary Figure 2A**.

### Molecular Information and Prediction Model Building

Molecular information, including the status of hormone receptor [estrogen receptor (ER), progesterone receptor (PR)], human epidermal growth factor receptor 2 (HER2), and Ki67 expression, was recorded from IHC results. ER/PR negative was defined as <1% of tumor cells with positive nuclear staining and ER/PR positive as ≥1% of tumor cells with positive nuclear staining; the cutoff for Ki67 was 14%; tumors with IHC staining of 0 or 1 were defined as HER2 negative, whereas tumors that either showed 3+ IHC staining or had gene copy number >2.0 were considered HER2 positive (23). The molecular-only LDA and MLP models were constructed by using the molecular information as input. The structure of the MLP neural network is shown in **Supplementary Figure 2B**.

### Radiomics Analysis and Image-Based Radiomics Analysis Prediction Model Building

For RA, the tumors were segmented on DCE-MRI images obtained 88 s after the beginning of the contrast agent injection, as the clinical breast DCE-MRI guideline indicates peak enhancement and obvious conspicuity at this time point in most breast cancers (24). Tumor segmentation was performed using ITK-SNAP software (https://www.itksnap.org) by one radiologist (ZC, with 10 years of experience in breast MRI) who was blinded to the clinical and histopathologic results. Tumors were segmented on a section-by-section basis until the whole tumor volume was captured and a three-dimensional ROI was acquired. A second radiologist (JS, with 21 years of experience in breast MRI) reviewed all the delineations to ensure correct segmentation. The segmented images were processed by using the open-source Python 3.7 (https://www.python.org.) and PyRadiomics toolkit to extract 851 radiomics features, including image intensity statistical, shape, texture, and wavelet features (**Supplementary Table S2**). A coarse-to-fine feature selection strategy was applied to reduce the dimension and avoid overfitting. Redundant features were removed according to the Spearman correlation coefficient, and then the optimal feature subsets (**Supplementary Table S3**) were selected using least absolute shrinkage and selection operator (LASSO) regression. The prediction models, based on optimal image features, were built by using the five machine learning classifiers [i.e., LDA, support vector machine (SVM), random forest (RF), AdaBoost, and Naive Bayes] to verify the performance of the classifiers to predict pCR successfully. Then, the optimal classifier was used to build the image-only and image-based RA model.

**Figure 2:**
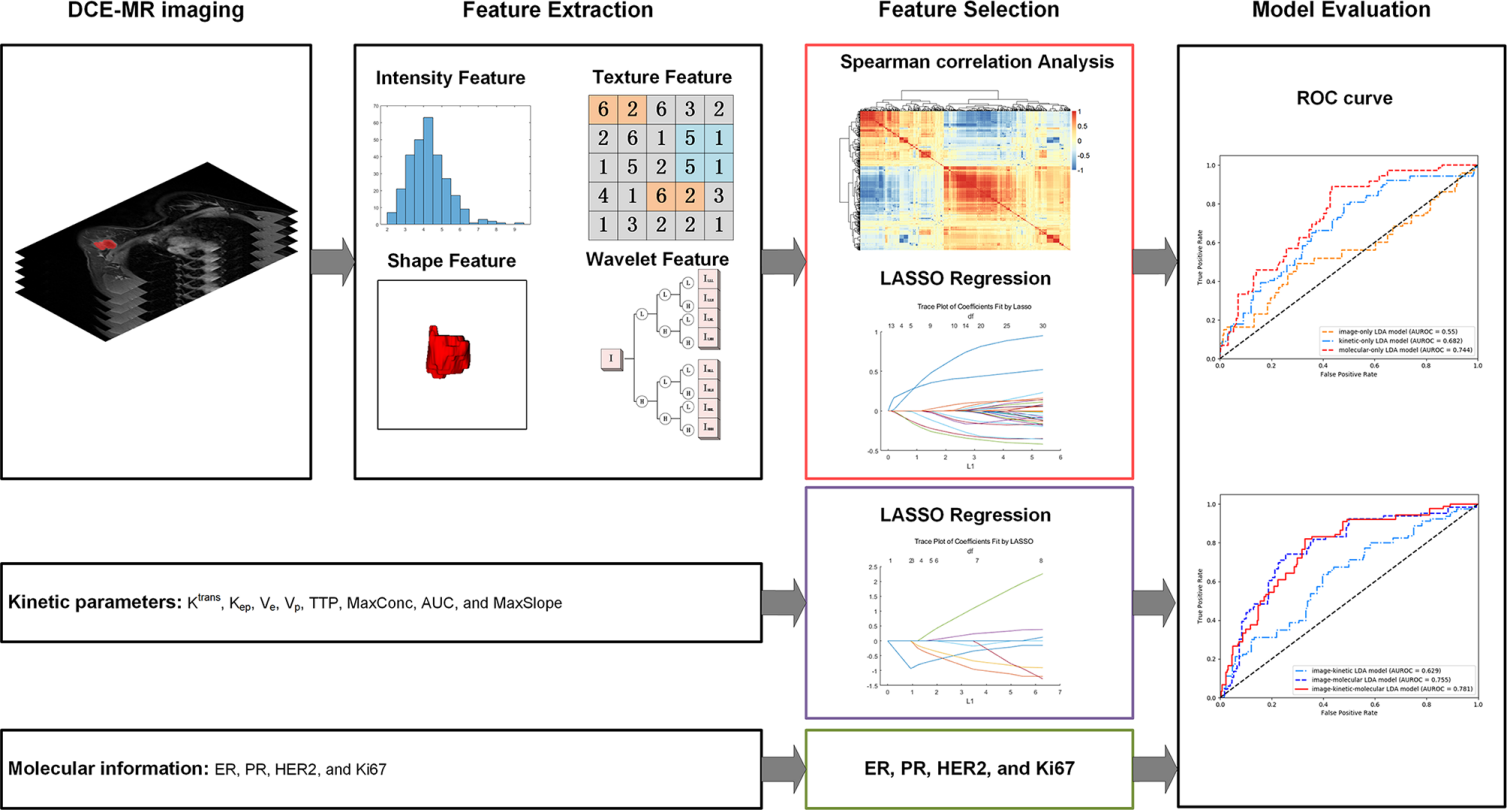
The workflow for building radiomics analysis-based predictive models.

The integrative image-based RA model was further developed by incorporating kinetic parameters (image-kinetic RA model), molecular information (image-molecular RA model), or both (image-kinetic-molecular RA model) into the image-only model. The optimal feature subsets of integrative image-based RA models are shown in **Supplementary Tables S4–S6**. The workflow for building RA predictive models is shown in **Figure 2**. All the RA models were constructed by using Matlab R2018b (MathWorks, Natick, MA, USA).

### Deep Learning Analysis and Image-Based Deep Learning Prediction Model Building

For DL analysis, a rectangular box of 128 × 128 × 3 pixels in size was used to crop three consecutive slices showing the maximum cross-sectional area of the tumor as input. To ensure comparability of the image signal intensity across patients, image intensity was normalized to a fixed range of 0–1. Random rotation, flip, and translation were used for data augmentation to alleviate the possible overfitting in the training procedure of model development. The image features were extracted by using a deep residual neural network, ResNeXt50 (25), pretrained on a large-scale, well-annotated ImageNet dataset to automatically learn discriminative image features, as illustrated in **Supplementary E3** and **Supplementary Figure S3**. The whole DL structure contained a ResNeXt50 CNN and three fully connected layers, with the probability of pCR as output to build the image-only CNN model. Adam optimizer was used to train all DL models with a learning rate of 0.0001 and a batch size of 32. The triplet loss procedure was introduced to extract more discriminative features using the output of ResNeXt50, and the cross-entropy was introduced as classification loss using the final output of the fully connected layer. Details of the loss function are provided in **Supplementary E4**.

**Figure 3:**
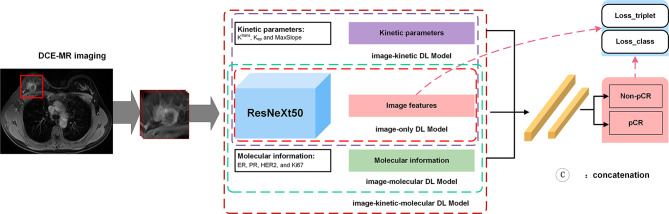
The framework for building deep learning-based predictive models.

The integrative image-based DL model was further developed by adding kinetic (image-kinetic DL model), molecular information (image-molecular DL model), or both (image-kinetic-molecular DL model) into the CNN of the image-only model. The kinetic and molecular information was incorporated in the first fully connected layer of DL models. The kinetic and molecular information was incorporated in the first fully connected layer of DL models. The framework for building DL predictive models is shown in **Figure 3**. All the DL programs were implemented in Pytorch (https://pytorch.org.) on an Intel Core i7-7700 K processor (Intel, Santa Clara, CA, USA) and Nvidia RTX 2080 Ti GPU with 11 GB RAM (Nvidia, Santa Clara, CA, USA).

### Statistical Analysis

Data were expressed as mean ± standard deviation for continuous variables, and categorical variables were summarized as frequencies and percentages. The differences in age, molecular information, histopathologic types, tumor number type, clinical T stage, clinical N stage, clinical TNM stage, and treatments between pCR and non-pCR groups were compared by χ^2^ or Wilcoxon rank-sum tests as appropriate. The inter-rater agreement of kinetic parameter evaluation was assessed by using the ICC. An ICC value >0.75 indicates good to excellent agreement. The predictive performance of the models was assessed by the ROC curve analysis. The sensitivity, specificity, positive predictive value (PPV), negative predictive value (NPV), and accuracy of the models were calculated based on a cutoff value determined by the maximum Youden index. And their confidence intervals were calculated by bootstrap analysis with 10,000-fold resampling. DeLong method was used to compare the AUROC between the models. A two-sided *P* value <0.05 indicated statistical significance. All statistical analyses were performed by using SPSS software (version 21; SPSS, Chicago, IL, USA) and MedCalc software (version 18.9.1; MedCalc, Ostend, Belgium).

## Results

### Clinicopathologic Characteristics

A total of 356 female patients (mean age, 46.9 ± 9.4 years) were included in this study. The clinicopathologic characteristics are shown in **Table 1**. Here, 83 patients (23.3%) achieved pCR (pCR group), while the remaining 273 patients (76.7%) were non-pCR (non-pCR group). pCR group had a higher prevalence of ER-negative, PR-negative, HER2-positive compared with the non-pCR group (all *P* < 0.001). There was no significant difference in age, Ki67, histological type, tumor number type, clinical T stage, clinical N stage, clinical TNM stage, breast surgery, and axillary surgery between the two groups (all *P* > 0.05).

**Table 1:** Clinicopathologic characteristics of patients in the non-pCR and pCR groups.

Characteristics	Non-pCR (n = 273)	pCR (n = 83)	*P*
Age (year)*	46.3 ± 9.4	48.2 ± 9.1	0.099
**ER status**			<0.001
Negative	68 (25)	49 (59)	
Positive	205 (75)	34 (41)	
**PR status**			<0.001
Negative	124 (45)	66 (80)	
Positive	149 (55)	17 (20)	
**HER2 status**			<0.001
Negative	187 (68)	27 (33)	
Positive	86 (32)	56 (67)	
**Ki67 status**			0.199
Negative	15 (5)	2 (2)	
Positive	258 (95)	81 (98)	
**Histological type**			0.601
IDC	255 (93)	80 (96)	
ILC	6 (2)	1 (1)	
Others	12 (5)	2 (2)	
**Tumor number type**			0.316
Single	224(82.1)	64(77.1)	
Multicentric and multifocal	49(17.9)	19(22.9)	
**Clinical T stage**			0.672
T1-2	154(56.4)	49(59.0)	
T3-4	119(43.6)	34(41.0)	
**Clinical N stage**			0.639
N0-1	242(88.6)	72(86.7)	
N2-3	31(10.9)	11 (13.3)	
**Clinical TNM stage**			0.920
I-II	153(56.0)	46(55.4)	
III	120(44.0)	37(44.6)	
**Chemotherapy**			<0.001
AT-based	217(79.5)	57(68.7)	
AC-based	38(13.9)	7(8.4)	
TC-based	18(6.6)	19(22.9)	
**HER2 positive therapy**			0.010
Trastuzumab	62(70.5)	28(49.1)	
Trastuzumab+pertuzumab	26(29.5)	29(50.9)	
**Surgery**			0.059
Mastectomy	100(36.6)	40(48.2)	
BCS	173(63.4)	43(51.8)	
**Axillary Surgery**			0.083
SLNB	63(23.1)	27(32.5)	
ALND	210(76.9)	56(67.5)	

Note: Unless indicated otherwise, values are numbers of patients with percentages in parentheses.

Abbreviations: pCR, pathological complete response; ER, estrogen receptor; PR, progesterone receptor; HER2, human epidermal growth factor receptor2; HR, hormone receptor; TNBC, triple-negative breast cancer; IDC, invasive ductal carcinoma; ILC, invasive lobular carcinoma; BCS, breast conserving surgery; SLNB, sentinel lymph node biopsy; ALND, axillary lymph node dissection; AT, anthracycline with paclitaxel; AC, anthracycline with cyclophosphamide; TC, paclitaxel with cyclophosphamide; TP, paclitaxel with platinum.

*Numbers are means ± standard deviations.

P values of the comparison between pCR and non-pCR patients in cohort were generated by one-way ANOVA for numerical variables and χ^2^ test for categorical variables.

### Image-, Kinetic-, and Molecular-Only Prediction Models

The LDA was the most robust classifier across multiple classifiers (**Supplementary Table S7**). The image-only LDA model had 12 image features selected by LASSO regression (**Supplementary Table S3**). The image-only CNN models had 1,000 image features extracted by ResNeXt50. The K^trans^, K_ep_, and MaxSlope were the independent predictors and included in the kinetic-only LDA and MLP models. Their AUROC, sensitivity, specificity, PPV, NPV, accuracy, and corresponding 95% CI are shown in **Table 2** and **Figures 4A, B**. The AUROC of the molecular-only LDA model was 0.744, which was higher than that of the kinetic-only LDA model (0.682, *P* = 0.012) and image-only LDA model (0.55, *P* < 0.001). The AUROC of the molecular-only MLP model was 0.752, which was higher than that of the kinetic-only MLP model (0.652, *P* = 0.007) and image-only CNN model (0.554, *P* < 0.001). The AUROC of the kinetic-only LDA model was 0.682, which was higher than that of the kinetic-only MLP model (AUROC = 0.652, *P* = 0.008). There was no significant difference between image-only LDA and image-only CNN models (AUROC = 0.55 and 0.554, *P* = 0.208), as well as between molecular-only LDA and molecular-only MLP models (AUROC = 0.744 and 0.752, *P* = 0.33).

**Table 2:** Performances of the image-, kinetic-, and molecular-only LDA and DL Prediction Models.

Model	LDA model			DL model		
	Image-only LDA model	Kinetic-only LDA model	Molecular-only LDA model	Image-only CNN model	Kinetic-only MLP model	Molecular-only MLP model
AUROC	0.55	0.682	0.744	0.554	0.652	0.752
	(0.513, 0.587)	(0.639, 0.726)	(0.688, 0.799)	(0.513, 0.595)	(0.612, 0.693)	(0.699,0.805)
Accuracy	0.58	0.638	0.673	0.558	0.65	0.663
	(0.502, 0.667)	(0.566, 0.711)	(0.617, 0.73)	(0.461, 0.656)	(0.592, 0.709)	(0.605,0.721)
Sensitivity	0.534	0.681	0.814	0.566	0.608	0.809
	(0.409, 0.660)	(0.546, 0.816)	(0.688, 0.939)	(0.392, 0.74)	(0.513, 0.703)	(0.682,0.936)
Specificity	0.6	0.625	0.632	0.556	0.663	0.619
	(0.465, 0.735)	(0.503, 0.748)	(0.541, 0.722)	(0.386, 0.726)	(0.575, 0.75)	0.527,0.712)
PPV	0.273	0.352	0.396	0.262	0.349	0.387
	(0.209,0.336)	(0.277, 0.427)	(0.322, 0.471)	(0.201,0.324)	(0.277, 0.422)	(0.313,0.461)
NPV	0.806	0.87	0.921	0.804	0.851	0.918
	(0.757,0.855)	(0.824, 0.915)	(0.874, 0.969)	(0.751,0.858)	(0.81, 0.892)	(0.869,0.966)
*P* ^*^	<0.001	0.012	–	<0.001	0.007	–
*P* ^#^	–	–	–	0.208	0.008	0.33

Note: Data in parentheses are 95% confidence intervals. LDA, linear discriminant analysis; MLP, multilayer perceptron; CNN, convolutional neural networks; DL, deep learning; AUROC, area under the receiver operating characteristics curve; PPV, positive predictive value; NPV, negative predictive value.

*P value of the comparison inside the LDA models and DL models, respectively.

# P value of the comparison between the LDA models and DL models, respectively.

**Figure 4:**
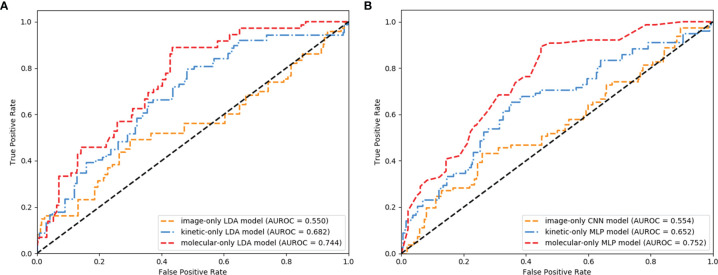
Receiver operating characteristic (ROC) curves of the image-, kinetic-, and molecular-only linear discriminant analysis (LDA) **(A)** and deep learning (DL) **(B)** models.

### Integrative Image-Based Radiomics Analysis and Deep Learning Models

**Figure 5:**
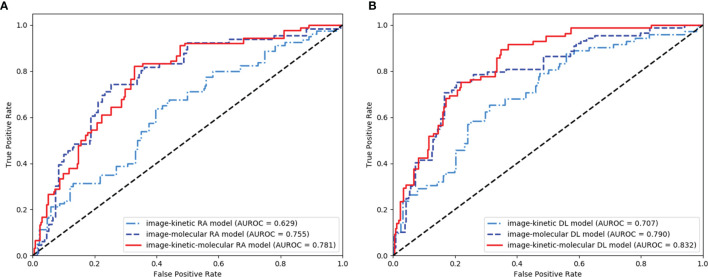
Receiver operating characteristic (ROC) curves of the integrative image-based radiomics analysis (RA) **(A)** and deep learning (DL) **(B)** models.

**Figure 6:**
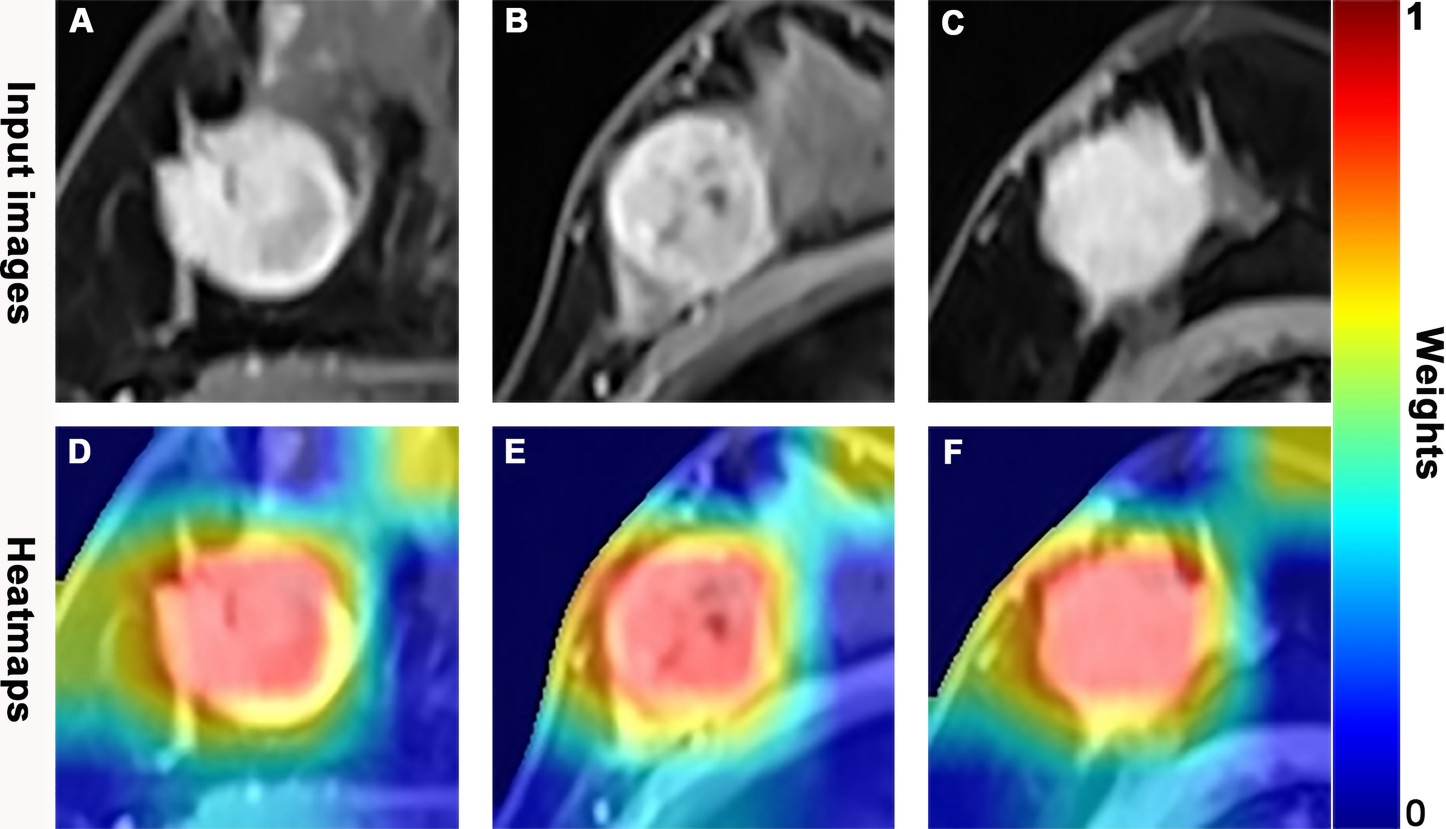
Dynamic contrast-enhanced magnetic resonance (DCE-MR) images and feature heatmaps generated from the ResNet50 in pathologic complete response (pCR) or non-pCR patients. The scaled weights of deep learning features are represented by the color bar. The color closer to red indicates that it has a greater weight and received more attention from the model. **(A, D)** A 41-year-old woman with an hormone response (HR)-positive/human epidermal growth factor receptor 2 (HER2)-negative invasive lobular carcinoma in the right breast and did not achieve pCR following 6 cycles of neoadjuvant chemotherapy (NAC). **(B, E)** A 53-year-old woman with a triple negative breast cancer (TNBC), invasive ductal carcinoma in the right breast, and achieved pCR following 8 cycles of NAC. **(C, F)** A 59-year-old woman with a HER2-positive invasive ductal carcinoma in the right breast and achieved pCR following 8 cycles of NAC.

The AUROC, sensitivity, specificity, PPV, NPV, accuracy, and corresponding 95% CI of integrative image-based RA and DL models are shown in **Table 3** and **Figures 5A, B**. The AUROC of the image-kinetic-molecular RA model was 0.781, which was higher than that of the image-kinetic RA model (0.629, *P* < 0.001), while it did not differ from the image-molecular RA model (0.755, *P* = 0.118). The AUROC of the image-kinetic-molecular DL model was 0.832, which was higher than that of image-kinetic and image-molecular DL models (0.707, 0.79; both *P* < 0.001). The heatmaps (**Figure 6**) generated from ResNeXt50 based on the Grad-Cam algorithm (26) indicated that locations were crucial in generating the output.

**Table 3:** Performances of the integrative image-based RA and DL models.

Model	RA model			DL model		
	Image-kinetic RA model	Image-molecular RA model	Image-kinetic-molecular RA model	Image-kinetic DL model	Image-molecular DL model	Image-kinetic-molecular DL model
AUROC	0.629	0.755	0.781	0.707	0.79	0.832
	(0.595, 0.663)	(0.708, 0.802)	(0.735, 0.828)	(0.654, 0.761)	(0.768, 0.812)	(0.816, 0.847)
Accuracy	0.619	0.695	0.731	0.661	0.752	0.772
	(0.571, 0.668)	(0.638, 0.753)	(0.678, 0.784)	(0.596, 0.725)	(0.715, 0.788)	(0.724, 0.821)
Sensitivity	0.647	0.778	0.795	0.692	0.797	0.781
	(0.559, 0.735)	(0.669, 0.887)	(0.703, 0.887)	(0.579, 0.806)	(0.723, 0.869)	(0.696, 0.867)
Specificity	0.611	0.671	0.712	0.65	0.739	0.769
	(0.537, 0.685)	(0.58, 0.762)	(0.634, 0.791)	(0.54, 0.761)	(0.681, 0.797)	(0.69, 0.849)
PPV	0.329	0.413	0.451	0.368	0.473	0.497
	(0.267, 0.391)	(0.333, 0.493)	(0.367, 0.536)	(0.318, 0.417)	(0.401, 0.546)	(0.408, 0.587)
NPV	0.855	0.911	0.922	0.88	0.925	0.924
	(0.816, 0.894)	(0.872, 0.951)	(0.888, 0.956)	(0.859, 0.902))	(0.897, 0.953)	(0.896, 0.953)
*P* ^*^	<0.001	0.118	–	<0.001	<0.001	–
*P* ^#^	–	–	–	<0.001	<0.001	<0.001

Note: Data in parentheses are 95% confidence intervals. RA, radiomics analysis; DL, deep learning; AUROC, area under the receiver operating characteristics curve; PPV, positive predictive value; NPV, negative predictive value.

*P value of the comparison inside the RA models and DL models, respectively.

# P value of the comparison between the RA models and DL models, respectively.

### Comparison Between Integrative Image-Based Radiomics Analysis and Deep Learning Models

The AUROC of image-kinetic, image-molecular, and image-kinetic-molecular DL model (0.707, 0.79, and 0.83, respectively) were significantly higher than that of the corresponding image-kinetic, image-molecular, and image-kinetic-molecular RA models (0.629, 0.755, and 0.781, respectively; all *P* < 0.001). The image-kinetic-molecular DL model had significantly higher AUROC than other integrative models (**Table 3**).

## Discussion

Our study results showed that both the molecular-only LDA and MLP models had a better prediction performance than the kinetic-only LDA and MLP model and image-only LDA and CNN model. The integrative image-kinetic-molecular RA and DL models significantly improved the predictive performance. Moreover, the image-kinetic-molecular DL model had the best performance (AUROC, 0.83) in predicting pCR before NAC in breast cancer patients.

Conventionally, the tumor size is used to assess the effect of NAC. Whereas the baseline tumor size cannot predict pCR (7, 10). It has been shown that molecular biomarkers are correlated with NAC sensitivity in breast cancer (27). For example, HR negativity and HER2 positivity were associated with higher pCR rates [odds ratio (OR) = 0.497 and 1.833, respectively] (28). The IHC4 score combining ER, PR, HER2, and Ki67 expression levels was associated with pCR rate; furthermore, the lower the IHC4 score, the higher the pCR rate in the ER-positive breast cancer patients (AUROC = 0.613) (29). Our results showed that the molecular-only LDA and MLP model achieved an AUROC of 0.744 and 0.752 in the breast cancer patients, higher than kinetic-only and image-only predictive models. However, the molecular information is acquired *via* invasive needle biopsy, which cannot reflect certain pathophysiological characteristics of tumors, such as microvascular density and permeability, and tumor heterogeneity, which is known to be relevant to the sensitivity of pCR NAC in breast cancer (15, 30).

The kinetic parameters can reflect the pathophysiological microvascular characteristics of tumors (6, 31). Previous studies (7–10) with a small sample size showed that pretreatment K^trans^, K_ep_, or V_e_, or their change after two cycles of NAC, could predict pCR but has a varying AUROC (0.658–0.93). More importantly, the metric capable of predicting pCR before NAC is more desirable in clinical settings. Identifying breast cancer patients who can truly benefit from NAC is crucial for successfully sparing toxicity and optimally selecting patients for endocrine or targeted therapy vs. chemotherapy. Whether the pretreatment value of K^trans^, K_ep_, or V_e_ could predict pCR remains to be determined. Our study showed that the kinetic-only LDA and MLP models building based on the pretreatment DCE-MRI achieved an AUROC of 0.682 and 0.656, comparable to the change of K^trans^, K_ep_, or V_e_ after two cycles of NAC (9, 10).

Breast cancer is a highly heterogeneous disease. The prediction performance of molecular-only and kinetic-only models was suboptimal for predicting pCR, and the highest AUROC of the molecular-only MLP model was only 0.752 in our study. The image features extracted from DCE-MRI could reflect spatial heterogeneity, including volumetric distribution of microvascular density and the extracellular compartment (32, 33). The image-only LDA and CNN models based on image features derived from pretreatment DCE-MRI were inadequate for predicting pCR (AUROC, 0.55 and 0.554). In theory, adding kinetic parameters or molecular information to the image-only model may improve predicting pCR to NAC. Indeed, the performance of the image-kinetic, image-molecular RA, and DL models (AUROC, 0.629 and 0.755; 0.707 and 0.79) was also undesirable. The integrative RA and DL models, including image features, kinetic parameters, and molecular information, improved the counterparts of model performance in predicting pCR to NAC with an AUROC of 0.781 and 0.83, which might represent more tumor heterogeneity comprehensively. Previous studies (12, 14) have also shown that the prediction performance of the RA or DL model based on pretreatment MRI in predicting pCR in breast cancer patients could be improved by combining with molecular information.

Notably, our results showed that the prediction performance of integrative DL models, including image-kinetic, image-molecular, and image-kinetic-molecular DL models was higher than that of the corresponding RA models. The image-kinetic-molecular DL model achieved the best performance (AUROC, 0.83) in predicting pCR before NAC. The most crucial aspect of DL, which significantly departs from radiomics classifiers, is that multiple and deep layers of perceptions capture low- to high-image features that are not designed by human engineers but are learned based on representation learning (11). Previous studies have also reported that the performance of DL is better than RA in breast lesion discrimination (17), axillary lymph node metastasis prediction (34), and esophagus cancer treatment prediction (35). In addition, unlike the radiomics feature extraction procedure, DL feature extraction only needs setting a bounding box of fixed size to the tumor region, which improves efficiency and offers more excellent reliability and higher reproducibility. For RA, handcrafted image segmentation is time-consuming and labor-intensive. Automatic and semiautomatic segmentation is less accurate for the lesions with low enhancement, indistinct or vague borders (i.e., diffuse non-mass enhancement), or the lesions in a moderate to marked background parenchymal enhancement (BPE) (36, 37). Taken together, the pretreatment DCE-MRI-based DL model in our study is clinically more favorable than the RA model for pretreatment prediction of pCR in breast cancer patients.

Our study has several limitations. First, the RA or DL approaches based on T2WI or DWI were not used to develop a prediction model. T2WI is not always able to clearly detect the exact border of breast cancer, especially in patients with dense breasts (38). In addition, DWI was easily affected by fat suppression and motion artifacts, which likely caused low reproducibility in ADC maps and ADC value (39). Previous studies have shown that RA or DL model established based on single T2WI, DWI, or ADC has relatively poor predictive ability (12, 16). Second, this study was a retrospective study in a single center. This may have caused selection bias. Third, the heterogeneous nature of molecular subtypes in breast cancer led to different NAC regimens and pCR probability, but this reflects the reality in clinical settings practice. Further investigation with multicenter and larger datasets is warranted to determine the generalization ability of our pretreatment DCE-MRI-based DL prediction model.

In conclusion, our study showed that the integrative image-based DL models are superior to the image-based RA models. The image-kinetic-molecular DL model achieved the best performance in predicting pCR to NAC in breast cancer patients.

## Data Availability Statement

The original contributions presented in the study are included in the article/**Supplementary Material**. Further inquiries can be directed to the corresponding authors.

## Ethics Statement

The studies involving human participants were reviewed and approved by Sun Yat-sen Memorial Hospital (Sun Yat-sen University, Guangzhou, China). The ethics committee waived the requirement of written informed consent for participation.

## Author Contributions

ZC and YP: guarantor of integrity of the entire study, study concepts, and design. ZC, YP, CG, CZ, XZ, and ZW: clinical studies and literature research. ZC, YP, and YY: statistical analysis. JS, JZ, and XY: article editing. All authors contributed to the article and approved the submitted version.

## Funding

This study was funded by the Key Areas Research and Development Program of Guangdong (Grant No. 2019B020235001) for JS, National Natural Science Foundation of China (Grant No. U1801681) for JS, Guangdong Province Universities and Colleges Pearl River Scholar Funded Scheme (2017) for JS, and Suzhou Science and Technology Bureau under Grant (SJC2021023) for JZ.

## Conflict of Interest

The authors declare that the research was conducted in the absence of any commercial or financial relationships that could be construed as a potential conflict of interest.

## Publisher’s Note

All claims expressed in this article are solely those of the authors and do not necessarily represent those of their affiliated organizations, or those of the publisher, the editors and the reviewers. Any product that may be evaluated in this article, or claim that may be made by its manufacturer, is not guaranteed or endorsed by the publisher.
