# Supplementary material for: Pretreatment DCE-MRI-Based Deep Learning Outperforms Radiomics Analysis in Predicting Pathologic Complete Response to Neoadjuvant Chemotherapy in Breast Cancer
Source: Front Oncol. 2022 Mar 10;12:846775. doi: 10.3389/fonc.2022.846775 (PMC8960929; doi:10.3389/fonc.2022.846775)
Supplement: Supplementary file 1 [file DataSheet_1.docx]

**Supplementary Material**

**Supplementary E1. Splitting into Training, Validation Set**

To effectively evaluate and compare the performance of machine learning algorithms, it is essential to split the cohorts into independent training and validation set. Most algorithms in medical use try to predict one entity (in our case NAC-induce pCR) based on the same patient data (in our case MRI data and molecular biomarkers). This is often done by “training” (i.e., adjusting the parameters of the algorithm) and potentially tweaking some training hyperparameters (such as how fast the algorithm should learn, or how many features should be selected). If the validation set contains samples of training set, which used to train the model, the model results will be overfitting and inaccurate, specifically: too optimistic. Accordingly, we strived to construct and evaluate models by dividing the cohorts into training and validation set in a five-fold cross-validation scheme. That means we repeatedly divide those tumors into training tumors and validation tumors. This is the approach that we took: Evaluate the performance of the algorithms in an outer five-fold cross-validation (CV) scheme and tune the hyper-parameters (e.g., number of features in radiomics or weight of triplet loss in deep learning) by using an inner five-fold CV scheme in training set. Concretely, the cohorts were divided equally into five subsets (D1, D2, D3, D4, and D5). In the training phase, D1, D2, D3, and D4 were used as the training set to train radiomics or deep learning models. In the testing phase, D5 was used as the validation set to obtain predictive probabilities. After re-initializing the models, the above training and validation phases were cycled four times in turn. Finally, the prediction probabilities of five independent validation sets were collected as a whole set and used to evaluate the model performance. The illustration of the five-fold cross-validation procedure was shown in the Supplementary Figure 1.

**Supplementary E2.** **The Regimens of Neoadjuvant** **Chemotherapy (NAC)**

Either anthracycline and paclitaxel, paclitaxel without anthracycline or anthracycline without paclitaxel was supplemented. The HER2 positive treatment regimen was also supplemented with trastuzumab and/or pertuzumab on a patient-by-patient basis, depending on disease severity.

**Supplementary E3. Details of ResNeXt50 Network Architecture**

The backbone network architecture chosen to extract image features is ResNeXt50, which is pretrained on the ImageNet dataset. The convolutional neural network contains a total of 50 layers with residual blocks, which reduce gradient disappearance in training and improve model performance. Each residual block has 32 groups of grouped convolutions, and a grouped convolution contains two 1×1 convolutions and one 3×3 convolution. The ResNeXt50 comprises the following layers: a 7×7 convolution, a 3×3 convolution, three residual blocks with an output depth of 256, four residual blocks with an output depth of 512, six residual blocks with an output depth of 1024, three residual blocks with an output depth of 2048 followed by an average pooling layer and a fully connected layer, as illustrated in Supplementary Figure 3.

**Supplementary E4:** **Loss Function of the Deep Learning-based CNN Models**

The loss function of the deep learning-based CNN model contained two parts: classification loss and triplet loss. The cross-entropy was used as the classification loss (Equation 1) and triplet loss (Equation 2) was added for better discriminability.

$Loss\_class=\sum_{i=1}^{m} y_{i}\cdot\log(f(x_{i}))$ (1)

Loss_class is the classification loss, m is the number of lesions, *y_i_* is the NAC response of breast lesions, and *f(x_i_)* is the probability predicted by the CNN model. $Loss\_triplet=\sum_{l,m,n} \max[\rho+||f_{R}(x_{l})-f_{R}(x_{m})||-||f_{R}(x_{l})-f_{R}(x_{n})||,0]$ (2)

Loss_triplet is the triplet loss, $\rho$ is the margin. L, m, and n represent indices of the anchor, positive of the anchor and negative of the anchor of triplet loss ($y_{l}=y_{m},y_{l}\neq y_{n}$), respectively. $f_{R}(.)$ are the image features extracted by ResNeXt50. The overall loss of deep learning-based CNN is as follows:

$Loss_{all}=Loss\_class+\lambda Loss\_triplet$ (3)

$\lambda$ is a hyper-parameter of triplet loss which was optimized by the results of the inner-loop five-fold cross-validation in the training set.

**Supplementary Figure 1. The illustration of the five-fold cross-validation procedure**

**
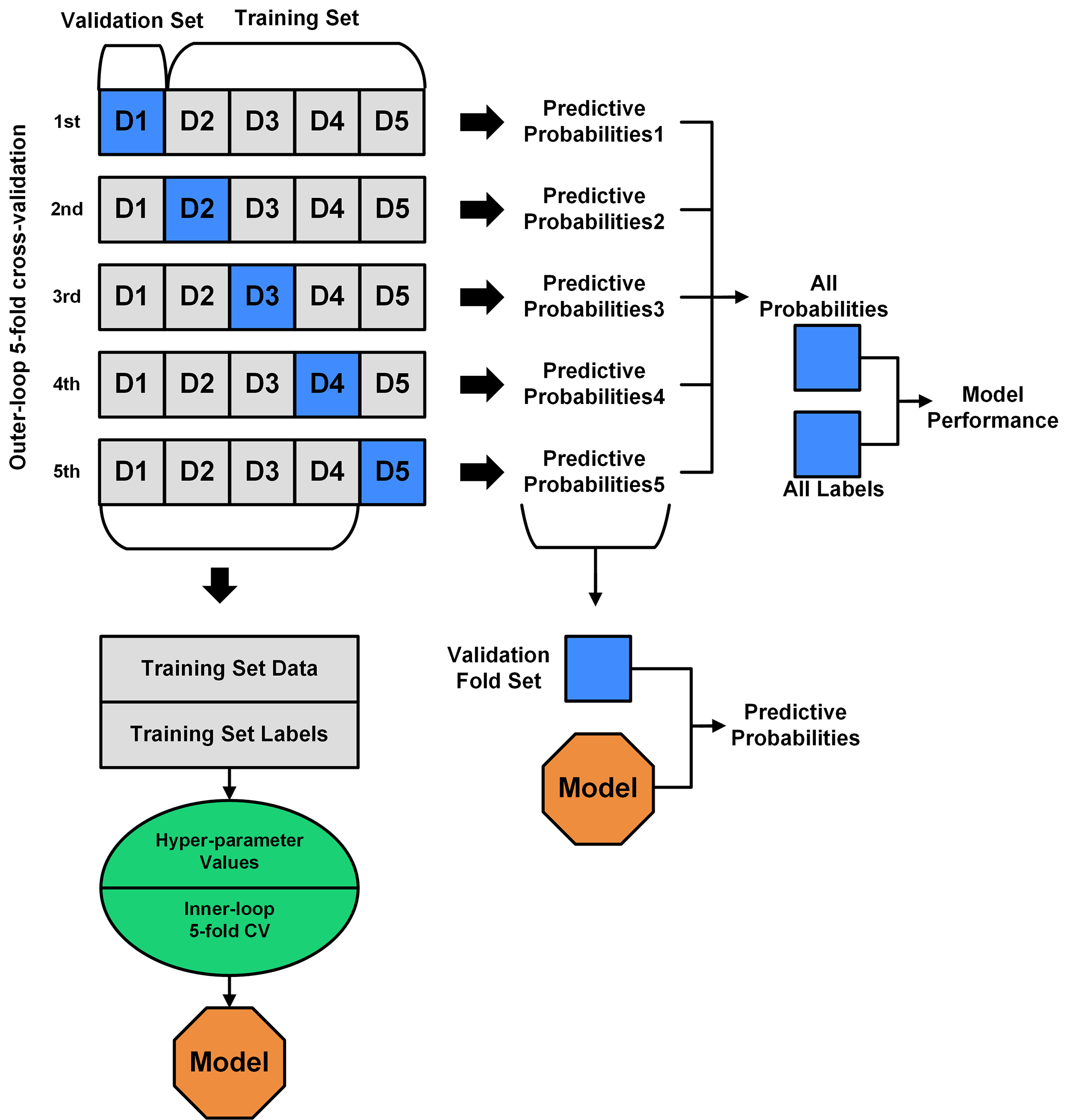
**

**Supplementary Figure 2. The structure of the MLP neural network**

**
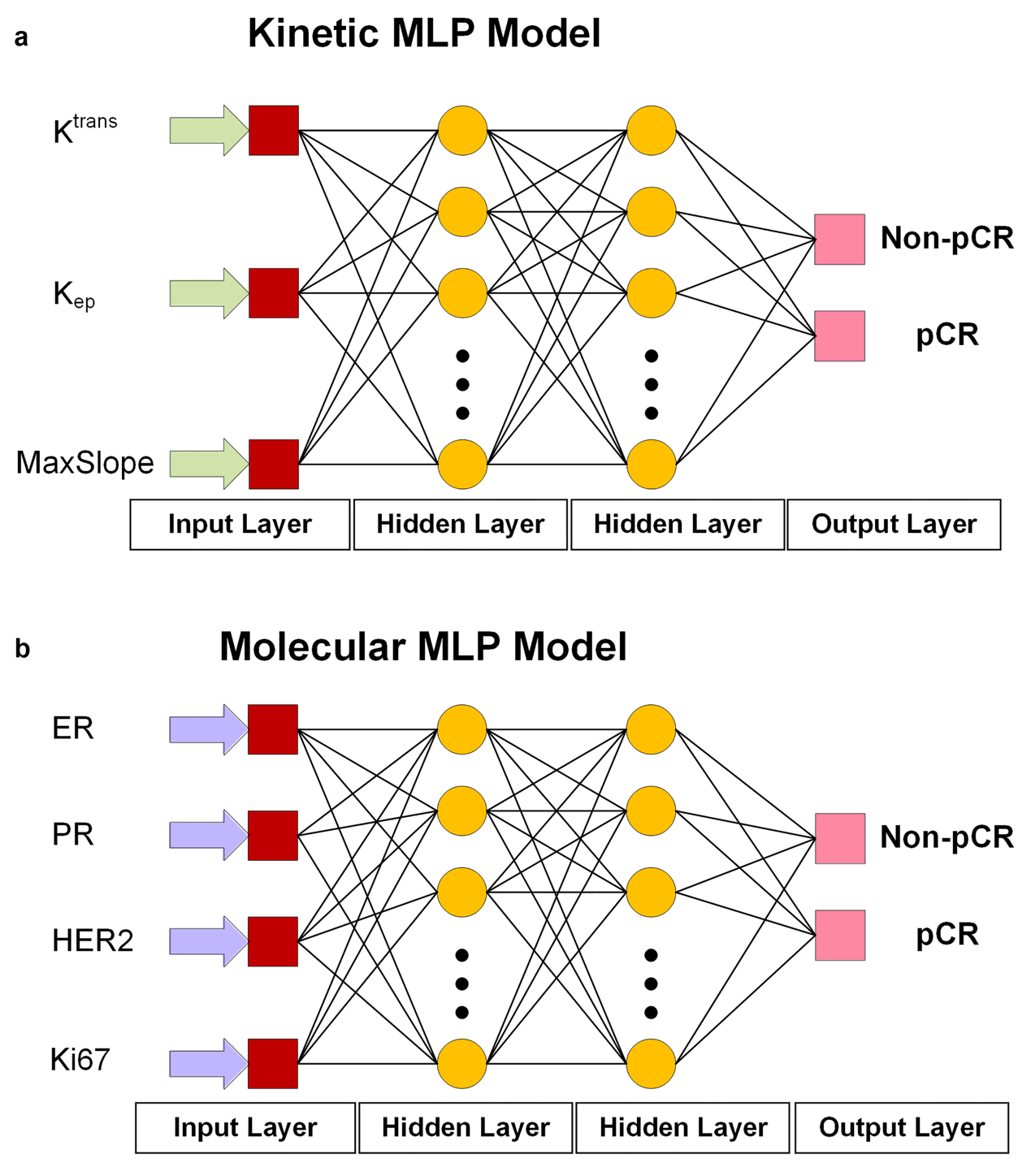
**

(a) The kinetic-only MLP model consists of one input layer with 3 neurons, according to the number of independent predictive kinetic parameters determined by LASSO, two hidden layers with 300 and 100 neurons, and one output layer with 2 neurons. (b) The molecular-only MLP model consists of one input layer with 4 neurons, according to the number of molecular parameters, two hidden layers with 300 and 100 neurons, and one output layer with 2 neurons. The output is the prediction of NAC treatment outcome with the status of pCR or non-pCR. The relationship between input and output is identified by calculating the weights in the neural network. Rectified linear units (ReLU) were used as an activation function in the MLP model, with the Softmax function as their classification function.

**Supplementary Figure 3. The structure of the ResNeXt50 Network**

**
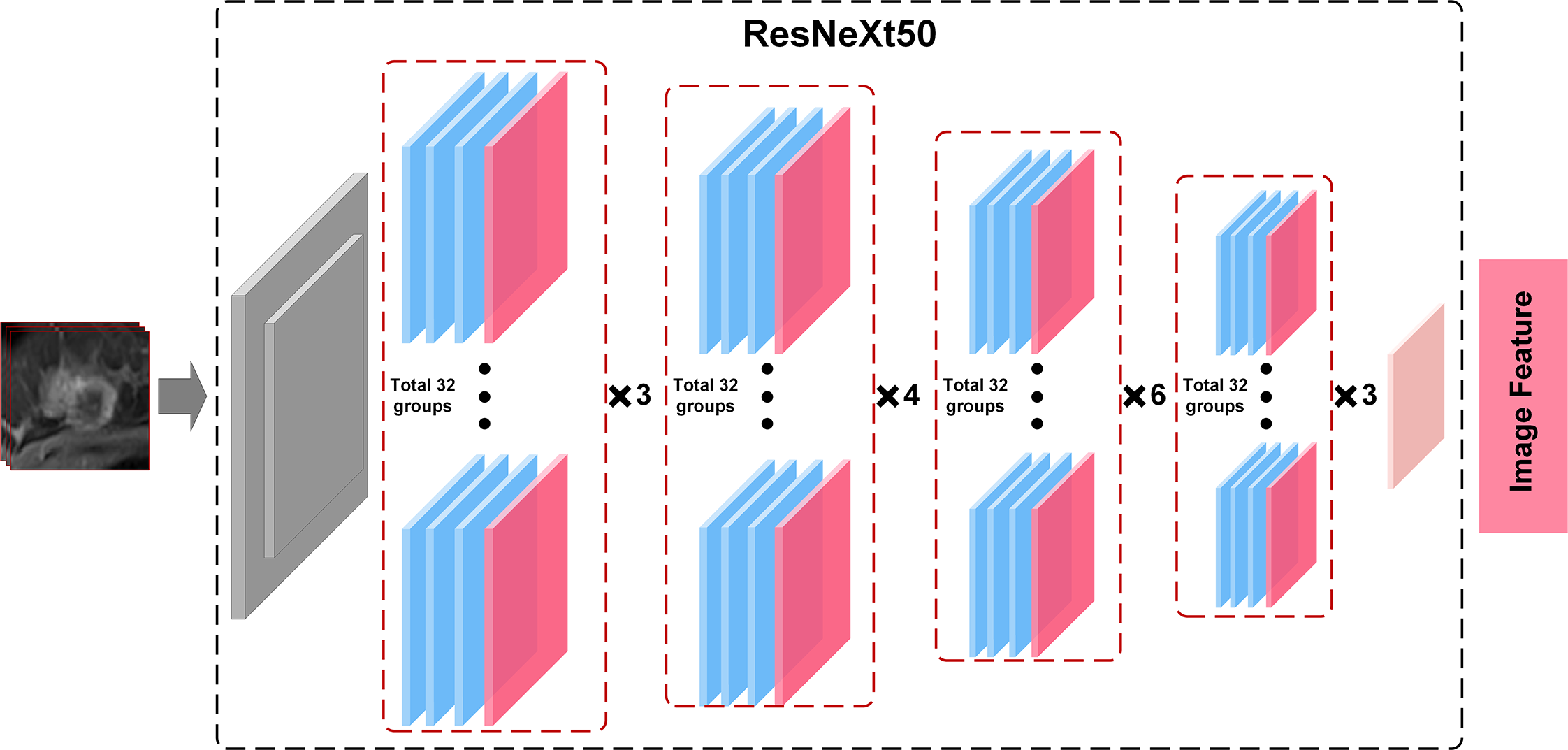
**

**Supplementary Table S1.** **Detailed acquisition parameters of MRI pulse sequence**

| MRI unit | Siemens 1.5T | | | |
| --- | --- | --- | --- | --- |
| Sequence | T2WI | T1WI-Dixon | DWI | T1WI-VIBE-FS  （DCE） |
| TR (ms) | 2760 | 6.86 | 5400 | 4.95 |
| TE (ms) | 107 | 2.39/4.77 | 119 | 2.2 |
| Flip angle (°) | 150 | 15 | 90 | 10 |
| FOV (mm) | 350×350 | 360×360 | 400×252 | 380×269 |
| Voxel size (mm) | 0.5×0.5×5.0 | 0.9×0.9×2.0 | 1.0×1.0×6.0 | 0.5×0.5×3.0 |
| Slice thickness (mm) | 5 | 2 | 6 | 3 |
| Overlapping gap (mm) | 1 | 0.2 | 1.8 | 0.6 |
| Temporal resolution (s) | / | / | / | 8 |
| Acquisition time (min) | 2′46″ | 2′20″ | 2′34″ | 5′-7′ |
| b values (s/mm²) | / | / | 0, 800 | / |

MRI=magnetic resonance imaging; DWI=Diffusion-weighted imaging; DCE=dynamic contrast-enhanced; TR=repetition time; TE=echo time; FOV=field of view; VIBE=volume interpolated body examination; FS=fat-suppression.

**Supplementary Table S2. Image features in the radiomics analysis**

| **Intensity statistical feature**  **(18 features)** | **Shape feature**  **(14 features)** | **Texture feature**  **(75 features)** | **Wavelet feature**  **(744 ((18 + 75) * 8) features)** |
| --- | --- | --- | --- |
| 10 percentiles | Elongation | GLCM (24 features) | Intensity statistical features and texture features of 8 wavelet filtered images |
| 90 percentiles | Flatness | GLSZM (16 features) |  |
| Energy | Least axis length | GLRLM (16 features) |  |
| Entropy | Major axis length | GLDM (14 features) |  |
| Interquartile range | Maximum 2D diameter column | NGTDM (5 features) |  |
| Kurtosis | Maximum 2D diameter row |  |  |
| Maximum | Maximum 2D diameter slice |  |  |
| Mean absolute deviation | Maximum 3D diameter |  |  |
| Mean | Mesh volume |  |  |
| Median | Minor axis length |  |  |
| Minimum | Sphericity |  |  |
| Range | Surface area |  |  |
| Robust mean absolute deviation | Surface volume ratio |  |  |
| Root mean squared | Voxel volume |  |  |
| Skewness |  |  |  |
| Total energy |  |  |  |
| Uniformity |  |  |  |
| Variance |  |  |  |

Note: The intensity statistical features describe the distribution of voxel intensities within the tumor region defined by the ROI mask. The shape features assess the whole tumor morphological characteristics. Texture features describe the variation of grayscale distribution inside and outside the tumor and are calculated with five different methods, including gray level co-occurrence matrix (GLCM), gray level size zone matrix (GLSZM), gray level run length matrix (GLRLM), gray level dependence matrix (GLDM), and neighboring gray-tone difference matrix (NGTDM). Wavelet features consist of intensity statistical features and texture features, which were extracted from the filtered DCE-MRI images by using wavelet filters. More details of all image features can be found online (<https://pyradiomics.readthedocs.io/en/latest/features.html>).

**Supplementary Table S3****.** **Radiomics features selected by LASSO for image-only RA model**

| Features | Feature type | pCR  (mean ± SD) | non_pCR  (mean ± SD) | *P* |
| --- | --- | --- | --- | --- |
| original_firstorder_10percentile | intensity | 2.401 ± 0.852 | 2.614 ± 0.827 | <0.001 |
| original_firstorder_Kurtosis | intensity | 2.851 ± 0.401 | 2.973 ± 0.599 | <0.001 |
| wavelet_LLH_GLDM_LargeDependenceLowGrayLevelEmphasis | wavelet texture | 310.212 ± 56.626 | 326.302 ± 46.567 | <0.001 |
| wavelet_LHL_GLCM_Imc1 | wavelet texture | -0.157 ± 0.022 | -0.155 ± 0.022 | <0.001 |
| wavelet_LHH_GLSZM_ZonePercentage | wavelet texture | 0.003 ± 0.003 | 0.002 ± 0.002 | <0.001 |
| wavelet_HLL_firstorder_90Percentile | wavelet intensity | 0.201 ± 0.075 | 0.181 ± 0.061 | 0.049 |
| wavelet_HLL_GLCM_Idn | wavelet texture | -0.145 ± 0.0144 | -0.142 ± 0.013 | <0.001 |
| wavelet_HLH_firstorder_Kurtosis | wavelet intensity | 5.005 ± 1.033 | 5.399 ± 1.333 | <0.001 |
| wavelet_HLH_NGTDM_Complexity | wavelet texture | 0.387 ± 0.015 | 0.389 ± 0.013 | <0.001 |
| wavelet_HHL_firstorder_Skewness | wavelet intensity | 0.090 ± 0.162 | 0.154 ± 0.208 | <0.001 |
| wavelet_LLL_GLSZM_SmallAreaHighGrayLevelEmphasis | wavelet texture | 0.056 ± 0.243 | 0.014 ± 0.064 | <0.001 |
| wavelet_LLL_NGTDM_Strength | wavelet texture | 0.044 ± 0.205 | 0.013 ± 0.079 | <0.001 |

Note: The selected image features between pCR and non-pCR were compared by using *t* test. LASSO=least absolute shrinkage and selection operator; The L or H in the “wavelet_LLH, LHL, LHH, HLL, HLH, HHL, and LLL” represents a Low or a High pass filter in each of the three dimensions of the wavelet filtering process. GLCM=gray level co-occurrence matrix; GLDM=gray level dependence matrix; GLRLM=gray level run length matrix; GLSZM=gray level size zone matrix; NGTDM=neighboring gray tone difference matrix.

**Supplementary Table S4.** **Radiomics features selected by LASSO for image-kinetic RA model**

| Features | Feature type | pCR  (mean ± SD) | non_pCR  (mean ± SD) | *P* |
| --- | --- | --- | --- | --- |
| original_firstorder_10 percentile | intensity | 2.401 ± 0.852 | 2.614 ± 0.827 | <0.001 |
| original_firstorder_Kurtosis | intensity | 2.851 ± 0.401 | 2.973 ± 0.599 | <0.001 |
| wavelet_LLH_GLDM_LargeDependenceLowGrayLevelEmphasis | wavelet texture | 310.212 ± 56.626 | 326.302 ± 46.567 | <0.001 |
| wavelet_LHL_GLCM_Imc1 | wavelet texture | -0.157 ± 0.022 | -0.155 ± 0.022 | <0.001 |
| wavelet_LHH_GLSZM_ZonePercentage | wavelet texture | 0.003 ± 0.003 | 0.002 ± 0.002 | <0.001 |
| wavelet_HLL_firstorder_90Percentile | wavelet intensity | 0.201 ± 0.075 | 0.181 ± 0.061 | 0.049 |
| wavelet_HLL_GLCM_Idn | wavelet texture | -0.145 ± 0.0144 | -0.142 ± 0.013 | <0.001 |
| wavelet_HLH_firstorder_Kurtosis | wavelet intensity | 5.005 ± 1.033 | 5.399 ± 1.333 | <0.001 |
| wavelet_HLH_NGTDM_Complexity | wavelet texture | 0.387 ± 0.015 | 0.389 ± 0.013 | <0.001 |
| wavelet_HHL_firstorder_Skewness | wavelet intensity | 0.090 ± 0.162 | 0.154 ± 0.208 | <0.001 |
| wavelet_LLL_GLSZM_SmallAreaHighGrayLevelEmphasis | wavelet texture | 0.056 ± 0.243 | 0.014 ± 0.064 | <0.001 |
| wavelet_LLL_NGTDM_Strength | wavelet texture | 0.044 ± 0.205 | 0.013 ± 0.079 | <0.001 |
| K^trans^ | kinetic parameters | 0.138 ± 0.105 | 0.209 ± 0.142 | 0.112 |
| K_ep_ | kinetic parameters | 0.927 ± 0.557 | 1.254 ± 0.775 | <0.001 |
| Max_Slope | kinetic parameters | 0.433 ± 0.203 | 0.500 ± 0.272 | <0.001 |

Note: The selected image features between pCR and non-pCR were compared by using *t* test. LASSO=least absolute shrinkage and selection operator; The L or H in the “wavelet_LLH, LHL, LHH, HLL, HLH, HHL, and LLL” represents a Low or a High pass filter in each of the three dimensions of the wavelet filtering process. GLCM=gray level co-occurrence matrix; GLDM=gray level dependence matrix; GLRLM=gray level run length matrix; GLSZM=gray level size zone matrix; NGTDM=neighboring gray tone difference matrix; K^trans^=volume transfer constant; K_ep_= reverse reflux rate constant; Max_Slope=maximal slope.

**Supplementary Table S5.** **Radiomics features selected by LASSO for image-molecular RA model**

| Features | Feature type | pCR  (mean±SD, frequency) | non_pCR  (mean±SD, frequency) | *P* |
| --- | --- | --- | --- | --- |
| original_firstorder_10 percentile | intensity | 2.401 ± 0.852 | 2.614 ± 0.827 | <0.001 |
| original_firstorder_Kurtosis | intensity | 2.851 ± 0.401 | 2.973 ± 0.599 | <0.001 |
| wavelet_LLH_GLDM_LargeDependenceLowGrayLevelEmphasis | wavelet texture | 310.212 ± 56.626 | 326.302 ± 46.567 | <0.001 |
| wavelet_LHL_GLCM_Imc1 | wavelet texture | -0.157 ± 0.022 | -0.155 ± 0.022 | <0.001 |
| wavelet_LHH_GLSZM_ZonePercentage | wavelet texture | 0.003 ± 0.003 | 0.002 ± 0.002 | <0.001 |
| wavelet_HLL_firstorder_90Percentile | wavelet intensity | 0.201 ± 0.075 | 0.181 ± 0.061 | 0.049 |
| wavelet_HLL_GLCM_Idn | wavelet texture | -0.145 ± 0.0144 | -0.142 ± 0.013 | <0.001 |
| wavelet_HLH_firstorder_Kurtosis | wavelet intensity | 5.005 ± 1.033 | 5.399 ± 1.333 | <0.001 |
| wavelet_HLH_NGTDM_Complexity | wavelet texture | 0.387 ± 0.015 | 0.389 ± 0.013 | <0.001 |
| wavelet_HHL_firstorder_Skewness | wavelet intensity | 0.090 ± 0.162 | 0.154 ± 0.208 | <0.001 |
| wavelet_LLL_GLSZM_SmallArea  HighGrayLevelEmphasis | wavelet texture | 0.056 ± 0.243 | 0.014 ± 0.064 | <0.001 |
| wavelet_LLL_NGTDM_Strength | wavelet texture | 0.044 ± 0.205 | 0.013 ± 0.079 | <0.001 |
| ER status | molecular  information | 68 (Negative) | 49 (Negative) | <0.001 |
|  |  | 205 (Positive) | 34 (Positive) |  |
| PR status | molecular  information | 124 (Negative) | 66 (Negative) | <0.001 |
|  |  | 149 (Positive) | 17 (Positive) |  |
| HER2 status | molecular  information | 187 (Negative) | 27 (Negative) | <0.001 |
|  |  | 86 (Positive) | 56 (Positive) |  |
| Ki67 status | molecular  information | 15 (Negative) | 2 (Negative) | 0.199 |
|  |  | 258 (Positive) | 81 (Positive) |  |

Note: The selected image features between pCR and non-pCR were compared by using *t* test. LASSO=least absolute shrinkage and selection operator; The L or H in the “wavelet_LLH, LHL, LHH, HLL, HLH, HHL, and LLL” represents a Low or a High pass filter in each of the three dimensions of the wavelet filtering process. GLCM=gray level co-occurrence matrix; GLDM=gray level dependence matrix; GLRLM=gray level run length matrix; GLSZM=gray level size zone matrix; NGTDM=neighboring gray tone difference matrix; ER=estrogen receptor; PR=progesterone receptor; HER2=human epidermal growth factor receptor2.

**Supplementary Table S6.** **Radiomics features selected by LASSO for image- kinetic-molecular RA model**

| Features | Feature type | pCR  (mean ± SD, frequency) | non_pCR  (mean ± SD, frequency) | *P* |
| --- | --- | --- | --- | --- |
| original_firstorder_10 percentile | intensity | 2.401 ± 0.852 | 2.614 ± 0.827 | <0.001 |
| original_firstorder_Kurtosis | intensity | 2.851 ± 0.401 | 2.973 ± 0.599 | <0.001 |
| wavelet_LLH_GLDM_LargeDependenceLowGrayLevelEmphasis | wavelet texture | 310.212 ± 56.626 | 326.302 ± 46.567 | <0.001 |
| wavelet_LHL_GLCM_Imc1 | wavelet texture | -0.157 ± 0.022 | -0.155 ± 0.022 | <0.001 |
| wavelet_LHH_GLSZM_ZonePercentage | wavelet texture | 0.003 ± 0.003 | 0.002 ± 0.002 | <0.001 |
| wavelet_HLL_firstorder_90Percentile | wavelet intensity | 0.201 ± 0.075 | 0.181 ± 0.061 | 0.049 |
| wavelet_HLL_GLCM_Idn | wavelet texture | -0.145 ± 0.0144 | -0.142 ± 0.013 | <0.001 |
| wavelet_HLH_firstorder_Kurtosis | wavelet intensity | 5.005 ± 1.033 | 5.399 ± 1.333 | <0.001 |
| wavelet_HLH_NGTDM_Complexity | wavelet texture | 0.387 ± 0.015 | 0.389 ± 0.013 | <0.001 |
| wavelet_HHL_firstorder_Skewness | wavelet intensity | 0.090 ± 0.162 | 0.154 ± 0.208 | <0.001 |
| wavelet_LLL_GLSZM_SmallAreaHighGrayLevelEmphasis | wavelet texture | 0.056 ± 0.243 | 0.014 ± 0.064 | <0.001 |
| wavelet_LLL_NGTDM_Strength | wavelet texture | 0.044 ± 0.205 | 0.013 ± 0.079 | <0.001 |
| K^trans^ | kinetic parameters | 0.138 ± 0.105 | 0.209 ± 0.142 | 0.112 |
| K_ep_ | kinetic parameters | 0.927 ± 0.557 | 1.254 ± 0.775 | <0.001 |
| Max_Slope | kinetic parameters | 0.433 ± 0.203 | 0.500 ± 0.272 | <0.001 |
| ER status | molecular  information | 68 (Negative) | 49 (Negative) | <0.001 |
|  |  | 205 (Positive) | 34 (Positive) |  |
| PR status | molecular  information | 124 (Negative) | 66 (Negative) | <0.001 |
|  |  | 149 (Positive) | 17 (Positive) |  |
| HER2 status | molecular  information | 187 (Negative) | 27 (Negative) | <0.001 |
|  |  | 86 (Positive) | 56 (Positive) |  |
| Ki67 status | molecular  information | 15 (Negative) | 2 (Negative) | 0.199 |
|  |  | 258 (Positive) | 81 (Positive) |  |

Note: The selected image features between pCR and non-pCR were compared by using *t* test. LASSO=least absolute shrinkage and selection operator; The L or H in the “wavelet_LLH, LHL, LHH, HLL, HLH, HHL, and LLL” represents a Low or a High pass filter in each of the three dimensions of the wavelet filtering process. GLCM=gray level co-occurrence matrix; GLDM= gray level dependence matrix; GLRLM=gray level run length matrix; GLSZM=gray level size zone matrix; NGTDM=neighboring gray tone difference matrix; K^trans^=volume transfer constant; K_ep_=reverse reflux rate constant; Max_Slope=maximal slope; ER=estrogen receptor; PR=progesterone receptor; HER2=human epidermal growth factor receptor2.

**Supplementary Table S7. The prediction performance of five machine learning classifier based on image features**

| Image-only | LDA | SVM | RF | AdaBoost | Naïve Bayes |
| --- | --- | --- | --- | --- | --- |
| model | model | model | model | model | model |
| AUROC | 0.55 | 0.538 | 0.522 | 0.531 | 0.51 |
|  | (0.513, 0.587) | (0.488, 0.588) | (0.479, 0.565) | (0.473, 0.589) | (0.478, 0.542) |
| Accuracy | 0.58 | 0.543 | 0.526 | 0.535 | 0.515 |
|  | （0.502, 0.667) | (0.443, 0.644) | (0.427, 0.626) | (0.428, 0.642) | (0.42, 0.61) |
| Sensitivity | 0.534 | 0.563 | 0.565 | 0.566 | 0.563 |
|  | （0.409, 0.660) | (0.388, 0.739) | (0.384, 0.745) | (0.387, 0.744) | (0.384, 0.742) |
| Specificity | 0.6 | 0.537 | 0.515 | 0.526 | 0.501 |
|  | (0.465, 0.735) | (0.365,0.709) | (0.34, 0.69) | (0.345, 0.707) | (0.33, 0.672) |
| PPV | 0.273 | 0.266 | 0.257 | 0.262 | 0.251 |
|  | （0.209,0.336） | (0.205, 0.327) | (0.202, 0.313) | (0.201, 0.324) | (0.20, 0.303) |
| NPV | 0.806 | 0.807 | 0.801 | 0.804 | 0.795 |
|  | （0.757,0.855） | (0.755, 0.859) | (0.749, 0.852) | (0.751, 0.858) | (0.746, 0.845) |

Note: AUROC=area under receive operating curve; LDA= linear discriminant analysis; SVM= support vector machine; RF=random forest; PPV=positive predictive value; NPV=negative predictive value
